# Supplementary figures and images for: Critical period plasticity is associated with resilience to short unpredictable stress
Source: Front Behav Neurosci. 2025 May 9;19:1584240. doi: 10.3389/fnbeh.2025.1584240 (PMC12098557; doi:10.3389/fnbeh.2025.1584240)

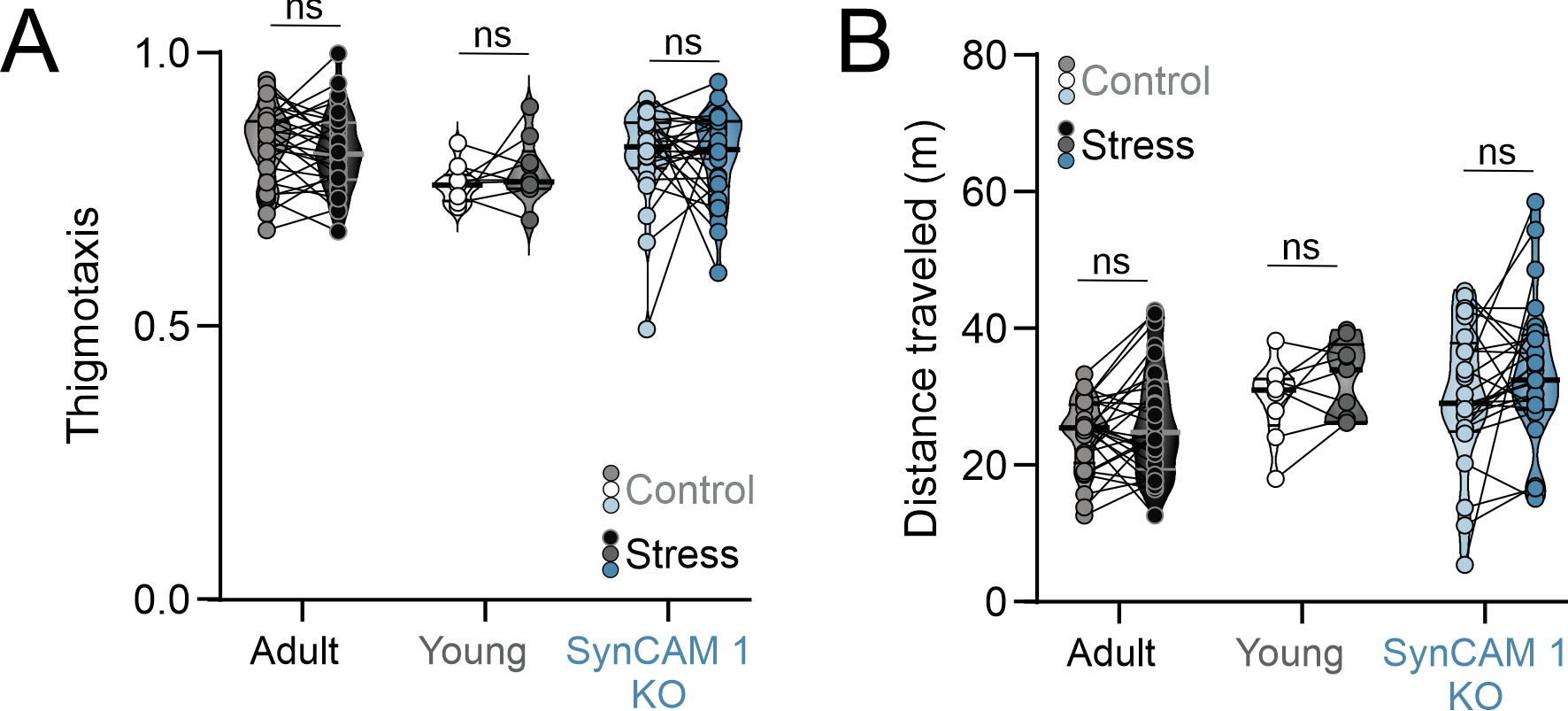

Supplement: Supplementary Figure 1 — Short unpredictable stress has no impact on anxiety-like behaviors. (A) Short unpredictable stress did not impact thigmotaxis in open field test. (B) While stressed mice from all groups travelled more in the open field arena, within-group post-hoc comparisons were not statistically significant. Medians and quartiles of the data are indicated by lines and individual points and lines represent mice. [file Image_1.png]
